# Serum ACE2 and S19P gene polymorphism in Egyptian patients with COVID-19 infection: correlation with disease severity

**DOI:** 10.1038/s41598-024-56260-6

**Published:** 2024-03-10

**Authors:** Dalia A. Gaber, Mohamed Shokr, Olfat Shaker, Kamelia Ahmed Zaki, Haidy Samir Khalil, Amany M. Wahb

**Affiliations:** 1 Medical Biochemistry and Molecular Biology Department, Faculty of Medicine, Helwan University, Cairo, Egypt; 2 College of Medicine, Gulf Medical University, Ajman, UAE; 3 Medical Biochemistry and Molecular Biology Department, Faculty of Medicine, MUST University, Cairo, Egypt; 4 Medical Biochemistry and Molecular Biology Department, Faculty of Medicine, Cairo University, Cairo, Egypt; 5 Medical Microbiology and Immunology, Faculty of Medicine, Helwan University, Cairo, Egypt

**Keywords:** Biochemistry, Immunology, Microbiology, Molecular biology

## Abstract

The expression of ACE2 is linked to disease severity in COVID-19 patients. The ACE2 receptor gene polymorphisms are considered determinants for SARS-CoV-2 infection and its outcome. In our study, serum ACE2 and its genetic variant S19P rs73635825 polymorphism were investigated in 114 SARS-CoV-2 patients. The results were compared with 120 control subjects. ELISA technique and allele discrimination assay were used for measuring serum ACE2 and genotype analysis of ACE2 rs73635825. Our results revealed that serum ACE2 was significantly lower in SARS-CoV-2 patients (*p =* 0.0001), particularly in cases with hypertension or diabetes mellitus. There was a significant difference in the genotype distributions of ACE2 rs73635825 A > G between COVID-19 patients and controls (*p*-value = 0.001). A higher frequency of the heterozygous AG genotype (65.8%) was reported in COVID-19 patients. The G allele was significantly more common in COVID-19 patients (*p < *0.0001). The AG and GG genotypes were associated with COVID-19 severity as they were correlated with abnormal laboratory findings, GGO, CXR, and total severity scores with *p < *0.05. Our results revealed that the ACE2 S19P gene variant is correlated with the incidence of infection and its severity, suggesting the usefulness of this work in identifying the susceptible population groups for better disease control.

## Introduction

The ACE2 peptidase is the predominant regulator of the renin–angiotensin–aldosterone system (RAAS)^1^. This enzyme catalyzes the conversion of angiotensin II to angiotensin-(1–7). The established axis then regulates normal body homeostasis and inhibits the pathological effects of the renin-angiotensin system^2^. On infection with SARS-COV-2, the viral cell entry is facilitated by ACE2 binding. ACE2 participates in SARS-CoV-2 entry by facilitating virus binding and fusion at the surface of susceptible cells^3^.

Acute lung inflammation results from lowered ACE2 levels, in addition to other accompanying pathologies including hypertension, type 2 diabetes, chronic renal failure, pulmonary diseases, and liver diseases^4,5^. This points to the role of SARS-CoV-2-induced ACE2 degradation in the severity of COVID-19^6–11^ and draws attention to the potential role of therapeutics that regulate the RAAS and ACE2/angiotensin-(1–7)/MAS in controlling organ injuries. The central role of ACE2 in both the effect on susceptibility to SARS-COV-2 infection and virus propagation as well as the effect of its expression level on the extent of inflammation has become a focus of research to understand mechanisms that can mitigate the disease severity^12^.

The ACE2 protein is abundant in various tissues of the body, including the lung, and colon with predominance in the kidneys, heart, and testicles^13^. The gene is located on chromosome Xp22^14^ and is considered a polymorphic gene with almost 140 single nucleotide polymorphisms (SNPs). Certain types of these SNPs are associated with COVID-19^15^.

The S19P rs73635825 variant was found to significantly affect the intermolecular binding between the receptor and S protein^16,17^. ACE2 gene expression levels vary among humans and are attributed to its gene variants created by SNPs in different populations^14^.

Studying the ethnic variations in such polymorphisms will explain the variable individual susceptibility to SARS-CoV-2 infection as well as it will explain the different outcomes of the disease. In the current era of personalized medicine, it is of utmost importance to investigate which gene variants are more common in severe cases since it will allow early screening and management^18^. S19P is an ACE2 SNP that was previously reported to affect ACE2 affinity towards SARS-CoV2 infection. However, the results of several studies were controversial whether they are associated with increased or decreased disease severity^19–21^.

Here, we designed our study to investigate the expression levels of ACE2 in Egyptian patients with SARS-Cov-2 infection and correlate them with the laboratory, clinical findings, and hence, infection severity. The S19P ACE2 SNP is also studied. The allele and genotype frequencies were investigated and correlated with clinical findings and the infection severity.

## Results

### Patients’ demographic data and laboratory findings

Sixty men and fifty-four women were included in this study. The patient’s age ranged between 32 and 88 years. 120 age- and sex-matched healthy controls (Seventy males and fifty females) were also enrolled in this study. No statistically significant difference was found between patients with SARS-CoV-2 and the controls regarding age and gender.

CBC revealed anemia in the infected group, manifested by decreased red cell count, decreased Hb concentration, and decreased hematocrit value which was significantly different from the control (*p =* 0.0001, 0.003, 0.035 respectively). The total leucocytic count was elevated with a significant difference from the control (*p =* 0.001). PT and INR values were also increased, with significant differences from controls (*p =* 0.0001) (Table 1).

**Table 1:** Laboratory findings in the studied groups.

Laboratory parameters	COVID-19 (N = 114)	Control (N = 120)	P-value
RBCs (× 10^6^/ µL)	4.30 ± 0.79	4.97 ± 0.05	0.0001*
Hb (g/dL)	11.77 ± 2.41	35.88 ± 7.02	0.003*
Hematocrit (%)	35.88 ± 7.02	38.90 ± 0.85	0.035*
MCV (fL)	83.36 ± 5.50	83.97 ± 4.74	0.395
MCH (pg)	27.26 ± 2.55	27.58 ± 1.97	0.461
MCHC (g/dL)	32.69 ± 1.36	34.29 ± 0.57	0.001*
Plts × 1000/µL	223.16 ± 87.39	290.75 ± 60.18	0.010*
TLC × 1000/µL	9.79 ± 5.90	5.89 ± 1.57	0.001*
PT	20.06 ± 7.30	11.48 ± 0.51	0.0001*
INR	1.44 ± 0.42	1.02 ± 0.033	0.0001*
Albumin (g/dL)	2.90 ± 0.50	4.45 ± 0.23	0.0001*
ALT (U/L)	62.74 ± 15.98	14.44 ± 5.22	0.002*
AST (U/L)	74.47 ± 15.95	17.36 ± 6.60	0.001*
LDH (IU/L)	515.90 ± 278.70	160.40 ± 25.57	0.0001*
Creatinine (mg/dL)	2.21 ± 2.05	0.67 ± 0.14	0.003*
Random Blood Sugar (mg/dL)	304.30 ± 146.26	132.72 ± 62.54	0.0001*
CRP (mg/dL)	45.01 ± 25.93	0	0.0001*
Plasma sodium (mEq/L)	138.23 ± 8.57	136.48 ± 1.63	0.312
PH	7.02 ± 0.76	7.31 ± 0.181	0.081
The partial pressure of carbon dioxide (PCO_2_)	44.41 ± 21.25	38.32 ± 6.19	0.002*
Partial pressure of Oxygen (pO_2_)	52.52 ± 21.01	88.32 ± 6.19	0.0001*
Bicarbonate (HCO_3_)	21.17 ± 6.70	23.09 ± 3.91	0.001*

Values are shown as mean ± SD, *p*-value. One-way ANOVA. *p* values in bold are statistically significant. *significant (*p* < 0.05).

Regarding the liver function tests, albumin was significantly lower in the SARS-CoV-2 patients with *p =* 0.0001, ALT, AST, and LDH were significantly elevated with *p =* 0.002, 0.001 & 0.0001 respectively. Other lab parameters were also altered in SARS-CoV-2 patients, as manifested by increased RBS, serum creatinine, and CRP with significant differences compared to control cases. Blood pH was lower in SARS-CoV-2 patients. ABG analysis revealed decreased pO_2_, increased pCO_2_, and decreased HCO_3_^-^ level with significant differences from controls (Table 1).

### Clinical-pathological findings in the SARS-CoV-2 patients

The range of hospital admission duration was 1–25 days and 87.7% were on oxygen therapy. 73.6% of cases were admitted to the ICU. Assessment of the associated comorbidities in SARS-CoV-2 patients revealed that 56.1% of the patients were hypertensive, and 81.6% were diabetic. Six patients had coronary diseases. Four patients had chronic kidney disease and were on hemodialysis. CT scan showed predominant ground glass opacities (GGO) in 21.9% of patients, consolidation in 5.3% and mixed GGO & consolidation in 56.1%. Pleural effusion was found in 32.5% of cases, nodal enlargement in 34.2% and pattern bronchial dilatation in 8.8% of patients.

According to the categorical CT assessment scheme (CO-RADS)^22^, patients were stratified based on the level of suspicion of SARS-CoV-2 infection into 8.8% low, 12.3% unsure, 26.3% high and 52.6% very high.

In the present study, we also assessed the patients according to the RSNA classification^23^. Results showed that 85.1% of the studied patients were considered typical, while the rest were undetermined. Based upon the GGO score, patients were classified into four different groups: mild; moderate; severe, and very severe. Also, according to the total severity score (TSS), 51.8% of the patients were diagnosed with severe SARS-CoV-2 infection with a score of mean ± SD of 10.83 ± 2.67, while 48.2% of the patients were non-severe with severity score 4.56 ± 2.150. There was a statistically significant difference between severe and non-severe patients with a p-value of 0.0001.

Chest X-ray (CXR) score was calculated to distinguish between patients. Patients were classified into four different groups: mild, moderate, severe, and very severe patients. The maximum score for the Modified Chest X-ray scoring system is 12. Mild patients' CXR score range between 1 and 4, the moderate group ranges between 5 and 8 while the severe patients' score range between 9 and 12, and very severe between 13 and 15^24^. Most of the patients were moderate patients with mean ± SD of CXR score 6.83 ± 0.90. The severe patients group covered about 28.9% of the total number of patients.

### ACE2 expression in SARS-CoV-2 patients

**Figure 1:**
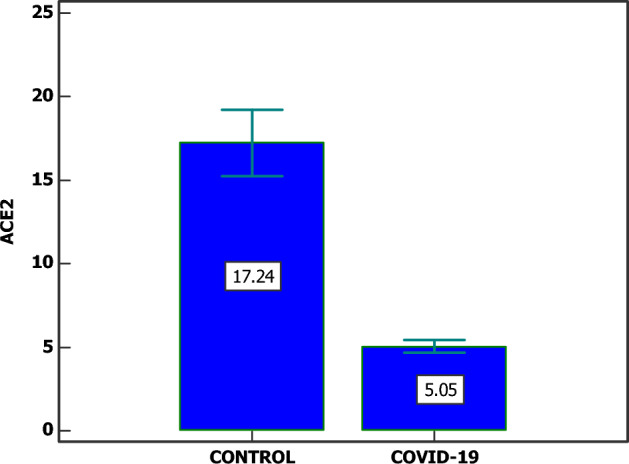
Expression level of ACE2 (U/L) among COVID-19 group and control group. SD is used, *significant (P < 0.05)*.

ACE2 expression was found to be lower in SARS-CoV-2 patients compared to the control group with a statistically significant difference (*p =* 0.0001) (Fig. 1). There was no correlation between the level of ACE2 and the age of patients.

### Correlations between ACE2 levels and clinical findings in SARS-CoV-2 patients

Correlations between serum level of ACE2 and clinical findings in SARS-CoV-2 patients revealed that the level of ACE2 was lower in patients with hypertension and/or diabetes mellitus but without significant difference. A statistical significance difference was noted in the serum ACE2 level between the typical (ACE2 = 4.88 ± 2.06 U/L) and undetermined groups (ACE2 = 6.0 ± 2.25 U/L) according to the RSNA classification with a p-value of 0.044. Patients with heart rates more than 100 bpm showed higher levels of ACE than the rest of the patients but without significant differences.

**Figure 2:**
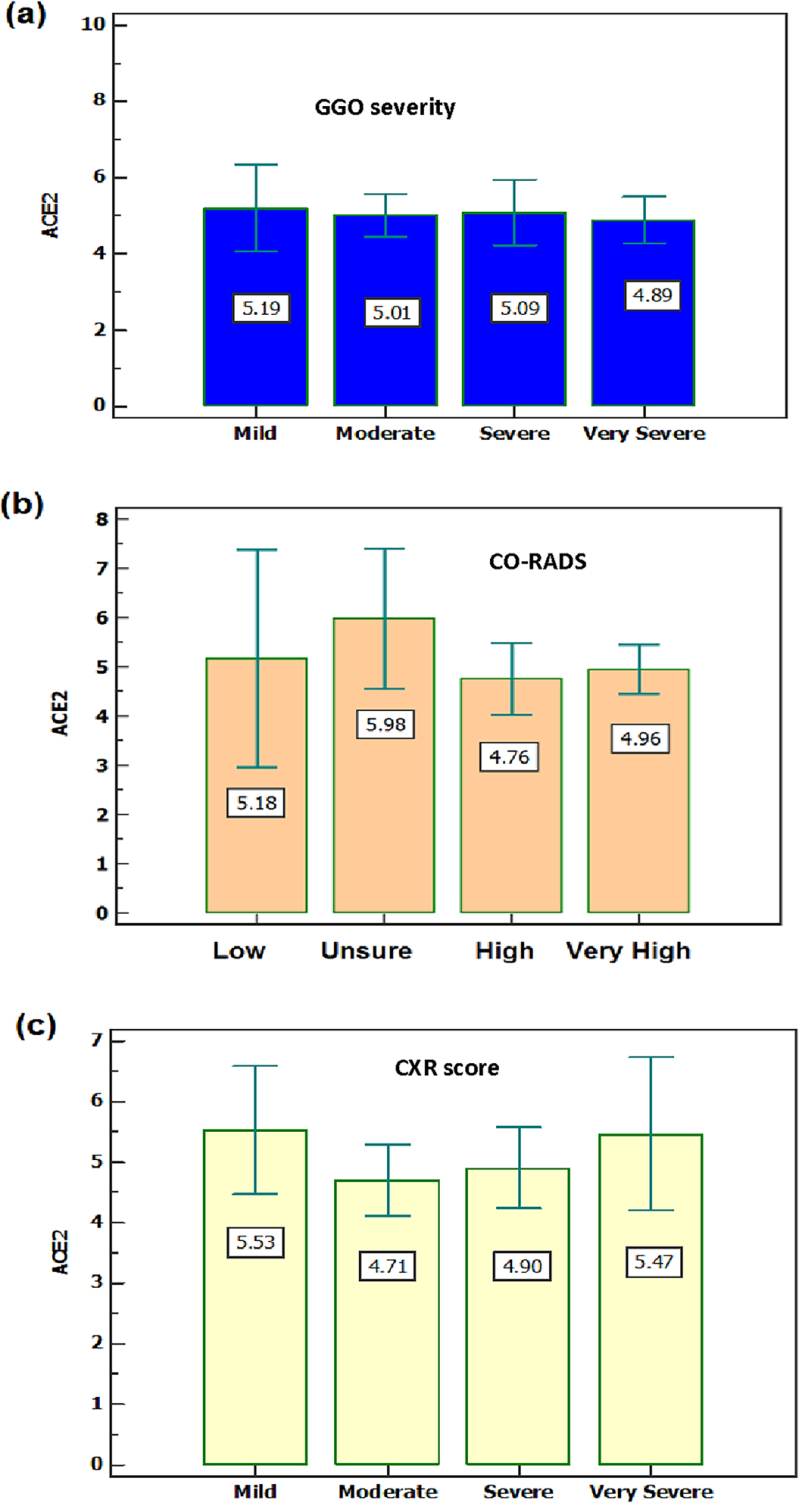
(**a**) Mean level of ACE2 as regards GGO severity; GGO- Ground-glass opacities 0–25 Mild,26–50 Moderate, 51–74 Severe, ≥ 75 Very severe. (**b**) Expression level of ACE2 as regards CO-RADS classification; mean ± SE is used. (**c**) Mean level of ACE2 as regards to CXR score; mild (score 1–4), moderate (score 5–8), and severe (score 9–12). SE is used. *significant (*p* < 0.05).

Comparing the TSS with the expression level of ACE2, no significant difference was detected between severe and non-severe patients with a *p*-value > 0.05. According to the GGO, the expression level of ACE2 tends to decrease in very severe patients than in other groups (Fig. 2a). According to the relationship between ACE2 levels and CO-RADS classifications among the COVID-19 patients group, results revealed that there were no significant differences between groups with a *p*-value > 0.05 (Fig. 2b). According to the CXR score, there were no statistical significance differences between mild, moderate, severe, and very severe patient groups as regards the expression level of ACE2 (*p* > 0.05) (Fig. 2c).

### Correlation between serum ACE2 and laboratory findings among SARS-CoV-2 patients

After measuring the bivariate correlation using Pearson r between serum ACE2 and laboratory findings among patients, results showed that there are no significant correlations found (Table 2).

**Table 2:** Correlations of ACE2 expression levels with laboratory findings in SARS-CoV-2 patient group.

Parameters	ACE2 r (*p*-value)
RBCs	− 0.078 (0.41)
Hemoglobin	− 0.051 (0.59)
Hematocrit (%)	− 0.053 (0.57)
MCV (fL)	0.064 (0.50)
MCH (pg)	0.051 (0.59)
MCHC (g/dL)	− 0.025 (0.79)
PLTs	− 0.055 (0.56)
WBCs	0.002 (0.98)
INR	0.056 (0.55)
Neutrophil	− 0.044 (0.64)
Lymphocyte	0.055 (0.56)
Monocyte	0.089 (0.34)
Basophil	0.061 (0.52)
Eosinophil	− 0.076 (0.42)
ALBUMIN	0.004 (0.97)
ALT	0.046 (0.62)
AST	0.046 (0.62)
Bilirubin	0.002 (0.98)
LDH	0.088 (0.35)
PT	0.064 (0.49)
Creatinine	0.003 (0.97)
Sodium (Na)	0.035 (0.71)
Potassium (K)	0.071 (0.45)
Random Blood Sugar	− 0.025 (0.79)
CRP	− 0.035 (0.71)
Heart Rate	0.047 (0.61)
The partial pressure of carbon dioxide (PCO^2^)	− 0.054 (0.56)
Partial pressure of Oxygen (pO^2^)	0.171 (0.07)
Bicarbonate (HCO^3^)	− 0.042 (0.65)

r Pearson correlation; *p* values in bold are statistically significant (< 0.05).

### Correlations between clinical scores in SARS-CoV-2 patients

Calculations of the correlations between the different clinical scores in SARS-CoV-2 patients showed that there was a positive correlation between CXR score and TSS with r = 0.32 and *p =* 0.001, between CXR score and CORADS with r = 0.34, *p =* 0.0002, between TSS and CORADS with r = 0.56, *p < *0.0001, between GGO percentage and CXR score with r = 0.242, *p =* 0.010, between GGO percentage and TSS with r = 0.892, *p < *0.0001 and, between GGO percentage and CORADS with r = 0.558, *p < *0.0001.

### Sensitivity and Specificity of ACE2 biomarker in the SARS-CoV-2 patient group

**Figure 3:**
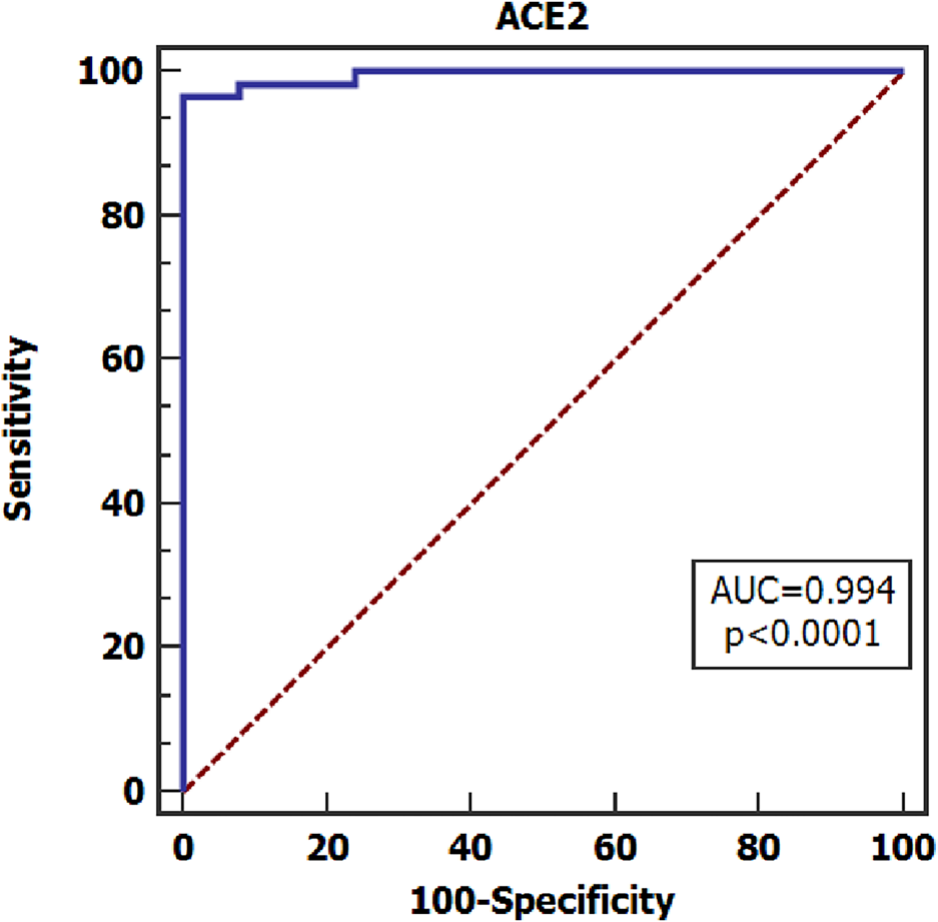
ROC Curve of ACE2 for SARS-CoV-2 patient group. Diagnostic performance of ACE2 for COVID-19, best cut off value = 6.37, sensitivity = 96.49%, specificity = 98.25%, accuracy = 97.37%, area under the curve (AUC) = 0.994, 95% confidence interval = 0.963 − 1.000 and *P < *0.0001.

According to the calculation of the result of biomarkers in serum samples from COVID-19 patients, we determined the applied value of ACE2 level as an indicator for diagnosis of COVID-19. Receiver Operating Characteristics (ROC) curves were carried out for analysis and showed that the cut-off value was 6.37, with a sensitivity of 96.49%, specificity of 98.25%, and accuracy of 97.37% (Fig. 3).

### Analysis of S19P rs73635825 polymorphism

“AA” is considered the reference genotype, comprising 95.8% of the healthy control group and 30.7% of the patient group. The majority of patients (65.8%) carried the the heterozygous genotype “AG” that varied significantly from the control group, *p =* 0.0001.

Only 3.5% of patients carried the homozygous variant “GG”, which was absent in the control group. Thus, the “G” allele was more common in the COVID-19 group with *p*-value of < 0.0001 (Table 3).

**Table 3:** Genotype distribution and allele frequencies of S19P.

S19P (rs73635825)	COVID-19	Control	p-value
Genotypes			
AA	35 (30.7%)	115 (95.8%)	0.0001*
AG	75 (65.8%)	5 (4.2%)	0.001*
GG	4 (3.5%)	–	**–**
Alleles			
A	145	232	0.0001*
G	83	8	< 0.0001*

*Significant at *p < *0.05.

Significant are in value [bold].

### Correlation between S19P genotypes and disease severity in COVID-19 patients

According to the GGO severity, results revealed that the “AG” genotype was associated with a moderate severity (GGO score of 48%) with *p =* 0.015.

Regarding chest x-ray score, the “AG” genotype was associated with the highest severity score followed by the “GG” genotype with a significant difference.

Based on the RSNA classification, the majority of patients who were classified as typical cases (57.9%) carried the “AG” genotype.

The highest total severity score 8.59 ± 3.61 was seen with the “AG” genotype with a significant difference from the “AA” & “GG” genotypes (Table 4).

**Table 4:** Association between S19P (rs73635825) genotypes and COVID-19 severity.

Parameter	S19P (rs73635825)			
	AA	AG	GG	*p*-value
GGO percentage	35.85 ± 22.41	47.93 ± 17.08	32.38 ± 23.89	**0.015**^**a**^
				0.82^b^
				0.085^c^
Chest X-ray score	6.83 ± 3.90	8.81 ± 3.47	8.50 ± 3.10	**0.007**^**a**^
				**0.005**^**b**^
				0.634^c^
Total severity score	6.37 ± 4.32	8.59 ± 3.61	5.75 ± 4.03	**0.005**^**a**^
				0.656^b^
				**0.006**^**c**^

^a^Between AA and AG, ^b^Between AA and GG, ^c^Between AG and GG.

Significant are in value [bold].

### Correlation between S19P genotypes (rs73635825) and laboratory findings of COVID-19 patients

The total WBC count was significantly higher in the “AG” genotype compared to the “AA” genotype with *p =* 0.014 and to the “GG” genotype with *p =* 0.013. Neutrophilia was predominant in the “AG” genotype with a significant difference from the “AA” genotype with *p =* 0.011. The other blood indices did not show significantly different values among the different genotypes.

CRP was relatively higher in the “GG” and “AG” genotypes. A significant difference was noted between the “GG” genotype and the “AA” genotype with *p =* 0.018 and between the “AG” genotype and the “AA” genotype with *p =* 0.017.

AST showed the highest elevation in the “AG” genotype with a significant difference from the “AA” group (*p =* 0.05) and from the “GG” genotype (*p =* 0.005). Plasma albumin was lower in the “AG” and “GG” genotypes with a significant difference from the “AA” genotype with *p =* 0.05 and 0.004 respectively. Serum bilirubin was higher in the “AG” and “GG” genotypes with significant difference from the “AA” genotype with *p =* 0.007 and 0.042 respectively. The “AA” genotype was associated with higher serum creatinine, *p =* 0.002 compared to the “AG” genotype and *p =* 0.003 compared to the “GG” genotype. Regarding the other laboratory tests, no significant difference was noted among the various genotypes (Table 5).

**Table 5:** Comparison between the laboratory findings of COVID-19 patients in genotypes of S19P (rs73635825).

Parameter	Genotypes in COVID-19 patients (*n* = 114) mean ± SD			
	S19P (rs73635825)			
	AA	AG	GG	p-value
Total_WBCs	7.74 ± 4.58	10.69 ± 6.21	9.62 ± 7.44	**0.014**^a^
				**0.033**^b^
				0.73^c^
Neutrophil	5.92 ± 3.25	8.53 ± 5.55	6.34 ± 2.13	**0.011**^a^
				0.57^b^
				0.32^c^
Lymphocyte	1.06 ± 1.0	1.16 ± 0.67	0.93 ± 0.38	0.58^a^
				0.88^b^
				0.33^c^
Monocyte	0.42 ± 0.13	0.43 ± 0.13	0.37 ± 0.07	0.78^a^
				0.12^b^
				0.81^c^
Basophil	0.014 ± 0.01	0.014 ± 0.01	0.023 ± 0.01	0.97^a^
				0.015^b^
				0.09^c^
Esinophil	0.014 ± 0.01	0.007 ± 0.01	0.003 ± 0.002	**0.048**^a^
				**0.028**^b^
				**0.18**^c^
INR	1.59 ± 0.44	1.37 ± 0.41	1.35 ± 0.17	**0.015**^a^
				**0.056**^b^
				**0.26**^c^
PT	22.08 ± 7.36	19.24 ± 7.33	19.07 ± 2.42	0.062^a^
				0.49^b^
				0.85^c^
D-dimer	0.83 ± 0.38	0.73 ± 0.44	0.75 ± 0.50	0.26^a^
				0.40^b^
				0.65^c^
CRP	36.31 ± 20.79	48.85 ± 27.04	49.50 ± 36.78	**0.017**^a^
				**0.04**^b^
				**0.018**^c^
ALT	73.51 ± 20.25	59.74 ± 10.08	27.75 ± 14.63	**0.049**^a^
				**0.002**^b^
				**0.0001**^c^
AST	66.91 ± 11.97	81.01 ± 13.61	25.0 ± 4.54	**0.05**^a^
				**0.001**^b^
				**0.005**^c^
Plasma Albumin	3.01 ± 0.39	2.82 ± 0.5	2.90 ± 0.87	**0.05**^a^
				**0.004**^b^
				0.06^c^
Serum Bilirubin	0.50 ± 0.17	0.74 ± 0.52	0.70 ± 0.14	**0.007**^a^
				**0.042**^b^
				0.27^c^
Serum Creatinine	2.89 ± 0.58	1.97 ± 0.24	1.02 ± 0.25	**0.002**^a^
				**0.003**^b^
				**0.001**^c^

^a^Between AA and AG, ^b^between AA and GG, ^c^between AG and GG.

Significant are in value [bold].

### Correlation between the serum ACE2 and S19P genotypes

**Figure 4:**
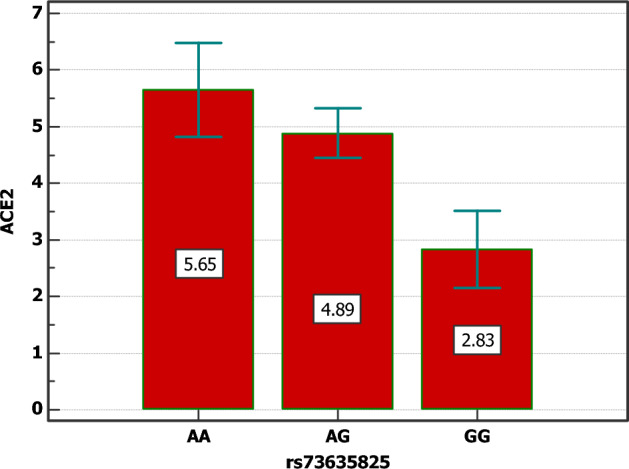
ACE2 expression level and S19P (rs73635825) genotype distribution among COVID-19 patients.

The lowest serum levels of ACE2 were noted among patients with the “GG” (2.83 ± 0.42 U/L) and the “AG” genotypes (4.88 ± 1.92 U/L) with significant differences compared to the “AA” genotype (*p =* 0.020) (Fig. 4).

## Discussion

Variations in the individual’s susceptibility to SARS-CoV-2 infection, as well as the differences among patients in the disease progression, have drawn researchers' attention during the COVID-19 pandemic. Infection incidence and severity are correlated with age, gender, ethnicity, and pre-existing health conditions. Genetic variations in the ACE2, which facilitates the virus entry into the cell, have become the center of attention in many studies^25^. The spike protein of SARS-CoV-2 binds to ACE2 facilitating receptor recognition and cell entry^26^. Currently, many studies are focused on unraveling more knowledge about ACE2 to plan strategies that can prevent and treat COVID-19^27^.

Our study was designed to investigate the ACE2 serum level and its genetic variant S19P rs73635825 polymorphism in SARS-CoV-2 Egyptian patients and relate it to the severity of infection and clinical status. Sixty men and fifty-four women with confirmed SARS-CoV-2 infection were included in this study as well as 120 age and sex-matched healthy controls. The recruited participants did not have any preexisting lung disease. The serum levels of ACE2 in patients were not affected by their gender. However, it had been demonstrated in a previous Chinese study^28^ that males were more prone to be infected by SARS-CoV-2 virus. The mentioned study linked this finding to the inhibitory effect of estrogen on ACE2 expression. Another study correlated the fact that the ACE2 gene is located on the X-chromosome with the higher incidence of infection among older men since they are hemizygous in terms of ACE2^29^. In agreement with our result, two studies^30,31^ showed that gender was not related to the incidence and severity of SARS-CoV-2. Statistical analysis in a former study showed that the severity of SARS-CoV-2 infection was more evident in older age group^32^. However, in our work, no statistically significant difference was found among patients with SARS-CoV-2 regarding age.

Complete blood picture (CBC), coagulation profile (including PT, INR, D-dimer), inflammatory biomarkers (including CRP, ESR, ferritin) as well as biochemical tests used to assess liver and kidney functions are routinely being requested for both diagnostic and prognostic purposes in COVID-19 as confirmed by many studies during the 2020 pandemic^33^. In our work, abnormalities in the laboratory investigations were evident in the infected group with significant differences from controls. CBC revealed anemia (decreased red cell count, decreased Hb concentration, and decreased hematocrit value), total leucocytic count was elevated, and coagulation profile showed prolonged prothrombin time and increased INR values. Plasma albumin was significantly lower in the SARS-CoV-2 patients, ALT, AST, and LDH were significantly elevated. Elevations in the random blood sugar and CRP were also noted. Blood pH was lower in SARS-CoV-2 patients. Arterial blood gases (ABG) analysis revealed decreased partial pressure of oxygen (pO_2_), increased partial pressure of carbon dioxide (pCO_2_), and decreased bicarbonate level with significant differences from controls.

Serum creatinine was significantly elevated in the patients group indicating kidney damage. It was suggested in previous studies that ACE2 is highly expressed in kidney tissues^34^ which facilitated the understanding of the mechanisms of abnormal renal function and kidney damage in COVID-19 patients. Also, ACE2 expression was studied in the rodent brain by immunohistochemistry techniques. It was found to be widely distributed in different areas of the brain and explained the neurological manifestations associated with COVID-19^35^. Analysis of the ACE2 gene variants revealed a high distribution of expression quantitative loci (eQTL) in different brain tissues, suggesting a possible link between ACE2 genetic variability and the neurological complications in patients with COVID-19^36^.

Based on our findings, serum ACE2 levels were significantly lower in SARS-CoV-2 patients compared to the control group. In patients with hypertension and/or diabetes mellitus, the serum ACE2 levels were even lower. In line with our results, Chen et al.^37^ reported that ACE2 expression significantly decreased in type 2 diabetic patients with COVID-19 infection. In another study^38^, the authors revealed that elevated plasma ACE2 levels in COVID-19 patients on hospital admission were associated with the worst disease outcomes. ACE2 levels were found to be even higher in hypertensive patients or those with pre-existing heart conditions as well as patients with kidney disease. Since viral cell entry is dependent on binding with ACE2, thus, high expression is expected to be correlated with increased infection rate and severity^36^. ACE2 facilitates viral cell entry and infection, then it becomes exhausted by the SARS-COV2 virus and its level starts to decline^7,8^.

The TSS stratified patients in our study into 51.8% severe and 48.2% non-severe. The serum ACE2 levels were significantly lower in typical and severe COVID-19 cases based on the RSNA classification and the TSS respectively. No significant correlations were found between serum ACE2 and the laboratory findings. The diagnostic value of ACE2 level as an indicator of COVID-19 was determined by ROC curve analysis. The cut-off value was calculated to be 6.37, with a sensitivity of 96.49%, specificity of 98.25%, and accuracy of 97.37%. In another study, the ACE1/ACE2 level was investigated in COVID patients and was found to have an important role in the pathogenesis of disorders associated with the renin-angiotensin system. A higher level of ACE1 was detected in ICU patients compared with non-ICU patients. The ACE1 rs1799752 polymorphism was associated with a high risk of SARS-CoV-2^39^.

The in-depth knowledge of variabilities in host genetics in a certain population may explain the different clinical responses to the virus as well and it will help in delivering effective measures to manage SARS-CoV-2 patients^40^. A former gene expression analysis study stated that the S19P gene variant of ACE2 was considered an interaction booster that facilitates the binding between the ACE2 receptor and the virus spike protein^15^. In our study, we assessed the S19P rs73635825 A > G polymorphism of the ACE2 gene and compared genotype and allele frequencies with the disease severity. So far and to our knowledge no studies have been conducted on ACE2 polymorphic genotype variants among Egyptian patients and their impact on the severity of COVID infection. Our genotype analysis revealed that the heterozygous “AG” constituted the main genotype in the patients’ group, while the reference “AA” genotype was present in the majority of the control group with significant difference between both groups. The “GG” variant was only detected among patients. The ACE2 genotype and allele distributions were in the Hardy–Weinberg equilibrium (HWE) for the healthy control group but it was slightly deviated in the diseased group. The same finding was noted in a previous study^41^ where the tested ACE genotypes slightly violated the HWE in the SARS-CoV-2-positive group. This finding was hypothetically attributed to an unrecognized selection bias. There was a statistically significant difference between the two groups regarding the S19P genotypes “AA” and “AG”. The “A” allele was more common in the control group, while the “G” allele was more common in the SARS-CoV-2 group. No significant difference was detected in comparing the S19P genotypes of SARS-CoV-2 patients’ group with age or gender. The “AG” genotype which was predominant in the patients’ group was found to be associated with a moderate disease severity based on the GGO score, the highest severity score for the chest x-ray classification, and presented the majority of typical cases based on the RSNA classification with significant differences from the other genotypes.

In another study that was conducted on SARS-CoV-2 patients in the Turkish population^31^, The ACE2 rs73635825 variant was very rare manifested by one patient carried this SNP, and no difference was observed between the groups. Another study conducted in Spain reported the increased risk and severity of COVID-19 in individuals who carry the ACE DD genotype of the ACE rs1799752 D/I polymorphism^41^, which came in contrast with a German study that demonstrated that the ACE rs1799752 D/I polymorphism had no effect on the severity or course of COVID-19 disease. The same study associated the G-allele of the ACE2 rs2285666 with an increased risk and more severe COVID-19 infection, with the A-allele significantly expressed in healthy people and associated with higher serum ACE2, while the GG genotype was associated with increased fatality rate^42^. It is noteworthy that such discrepancies among studies involving different populations are unclear and cannot be quite explained as declared by many authors.

The laboratory findings in the SARS-CoV-2 patients were compared with the various S19P genotypes. The total WBC count was significantly higher in the “AG” genotype compared to the other genotypes with significant differences. Neutrophilia was predominant in the “AG” genotype with a significant difference from the “AA” genotype. The other blood indices did not show significantly different values among the genotypes. CRP was relatively higher in the “GG” and “AG” genotypes with significant differences from the “AA” genotype. AST showed the highest elevation in the “AG” genotype with a significant difference from the other two genotypes.

Plasma albumin was lower in the “AG” and “GG” genotypes with a significant difference from the “AA” genotype. Serum bilirubin was higher in the “AG” & “GG” genotypes with significant differences from the “AA” genotype. The “AA” genotype was associated with higher serum creatinine, compared to the “AG” and “GG” genotypes. Regarding the other laboratory tests, no significant difference was noted among the various genotypes.

The lowest serum levels of ACE2 were noted among patients with the “GG” and the “AG” genotypes with significant differences compared to the “AA” genotype. It is crucial to mention that our study does not reflect the whole Egyptian population as further studies need to be conducted comprising a larger group. Also, since it is limited to one population, it cannot be transferred to other populations with different ethnicities. Our work shows that the ACE2 S19P rs73635825 polymorphism impacts the risk for SARS-CoV-2 infection and the disease severity.

## Conclusion

- The serum level of ACE2 was significantly lower in patients with SARS-CoV-2 compared to healthy controls. In addition, patients with co-morbidities showed lower ACE2 levels among other COVID-19 cases.
- Serum ACE2 can be used as an indicator of SARS-CoV-2 infection with a sensitivity of 96.49%, specificity of 98.25%, and accuracy of 97.37%. It can also be used as a prognostic indicator as it is correlated with the infection severity.
- A significant difference was noted in the ACE2 rs73635825 S19P genotype distribution between COVID-19 patients and controls. The heterozygous AG genotype was predominant among patients. Both AG and GG genotypes were associated with COVID-19 severity as they were correlated with abnormal laboratory and radiological findings.
- It can be concluded that ACE2 S19P genotype analysis can be useful in identifying the susceptible population groups for proper disease management.
- The limitation of the study includes a relatively small sample size of 234 participants.

## Patients and methods

### Study design

This prospective, cross-sectional study was conducted between January 2022 and February 2023 in the Departments of Medical Biochemistry and Molecular Biology, Faculty of Medicine, Helwan University and Cairo University. The study included 234 subjects classified into two groups:

- Group I: 114 patients with SARS-CoV-2 infection
- Group II: 120 healthy controls

### Patient selection

One hundred and fourteen patients who were admitted to the internal medicine department, quarantine hospital, Kasr Al-Ainy Faculty of Medicine, Cairo University participated in this study after being diagnosed with SARS-CoV-2 infection. 120 age- and sex-matched control specimens were obtained during blood sampling that was part of routine lab workups and were selected based on a negative PCR test. Demographic data and medical history were recorded for each patient. Informed consent was obtained from all participants.

### Inclusion criteria

- Patients with a positive nucleic acid amplification test (NAAT) for SARS-CoV-2. NAAT is considered the most sensitive test for detecting SARS-CoV-2 infection as recommended by the Centers for Disease Control and Prevention (CDC). https://www.covid19treatmentguidelines.nih.gov/overview/sars-cov-2-testing/
- Patients with mild, moderate, and severe SARS-CoV-2 illness, based on the USNIH (National Institutes of Health) clinical spectrum of SARS-CoV-2 infection.

### Exclusion criteria

- Patients suspicious of COVID-19 infection but with a negative NAAT.

### Sample size calculation

Sample size was calculated using MedCalc version 12.6 (MedCalc Software, Mariakerke, Belgium), and SAS 9.4 (SAS Institute Inc., Cary, NC) was used for statistical analysis.

Based on a previous study^43^, Type I error (0.05) and Type II error (0.20). The ratio of sample size in COVID patients and healthy individuals was 1. The total sample size was 134 (67 patients and 67 controls) as calculated by the Research Support Center, Helwan University, Cairo, Egypt. However, in our study, we had 234 participants (114 patients and 120 controls) for better result accuracy.

### Determination of disease severity

According to the WHO, patients were diagnosed with COVID-19 infection based on their clinical status and a positive PCR test. For stratification, patients were evaluated by chest imaging (X-ray, CT scan), and laboratory tests including a complete blood count with differential, liver and renal function test, and inflammatory markers like CRP.

The participants in this study were assessed by multiple expert evaluators after they were admitted to the internal medicine department, quarantine hospital, Kasr Al-Ainy Faculty of Medicine, Cairo University. The total severity score (TSS) was also calculated from the CT scan by expert radiologists.

Individuals with no clinical confirmation of the infection accompanied by a negative PCR test, were considered as the healthy control group.

The disease severity was determined based on the US NIH guidelines (https://www.covid19treatmentguidelines.nih.gov/overview/clinical-spectrum/) that classified the clinical Spectrum of SARS-CoV-2 infection into:

- Asymptomatic or Pre-symptomatic Infection: Individuals who test positive for SARS-CoV-2 using a virologic test (i.e., a nucleic acid amplification test [NAAT]) but who have no symptoms that are consistent with COVID-19.
- Mild Illness: Individuals who have any of the various signs and symptoms of COVID-19 (e.g., fever, cough, sore throat, malaise, headache, muscle pain, nausea, vomiting, diarrhea, loss of taste and smell) but who do not have shortness of breath, dyspnea, or abnormal chest imaging.
- Moderate Illness: Individuals who show evidence of lower respiratory disease during clinical assessment or imaging and who have an oxygen saturation (SpO_2_) ≥ 94% on room air at sea level.
- Severe Illness: Individuals who have SpO_2_ < 94% on room air at sea level, a ratio of arterial partial pressure of oxygen to fraction of inspired oxygen (PaO_2_/FiO_2_) < 300 mm Hg, a respiratory rate > 30 breaths/min, or lung infiltrates > 50%.
- Critical Illness: Individuals who have respiratory failure, septic shock, and/or multiple organ dysfunction.

To assess statistical risk, the study subjects were divided into two categories: serious and non-serious patients. The non-serious group included asymptomatic, mild, and moderate cases, while the serious group consisted of all severe and critical patients who were admitted to the hospital, whether inwards or in the ICU.

### Sample collection

Whole blood samples (10mL) were collected in sterile vacutainers from patients diagnosed with SARS-CoV-2 at the internal medicine department, quarantine hospital, Kasr Al-Ainy Faculty of Medicine, Cairo University. The samples were centrifuged at 4000 rpm for 10 min for serum isolation and the serum was stored at − 80 ºC to be used for quantitative assessment of serum ACE2. 5 ml of whole blood were collected in EDTA tubes for DNA extraction.

### Measurement of ACE2 expression

Quantitative determination of serum human ACE2 was done using an ELISA kit, provided by Bioassay Technology Laboratory (Zhejiang, China), Cat. No E3169Hu.

### Other laboratory investigations

Complete blood picture (CBC hematology XN-350 analyzer, Sysmex, Japan), coagulation profile (BCS XP, Sysmex, Japan), liver function tests, serum creatinine, random blood sugar, CRP (AU680 Clinical Chemistry Analyzer, Beckman coulter, USA), blood pH, partial pressure of carbon dioxide (PCO_2_), and oxygen (PO_2_), serum potassium and serum bicarbonate (Fully Automatic Gem Premier 3000 Blood Gas Analyzer, IL Werfen, Spain) were done for all the patients.

#### DNA extraction

DNA is purified from whole blood with a Qiagen DNA extraction kit (Hilden, Germany) following the manufacturer's protocol.

### Genotyping assay

Genotype distributions of the S19P SNPs (rs73635825) of the ACE2-receptor are detected by allele discrimination assay utilizing TaqMan probes (Applied Biosystems, USA). The TaqMan probes are labeled with VIC and FAM fluorescent dyes.

Sample processing and biosafety measures were done following the lab safety guidelines and took approval no. MS-44-2021.

### Research ethics statement

This study was approved by the Research Ethics Committee of the Faculty of Medicine, Helwan University, Egypt [Serial No.69-2022]. An informed consent was obtained from each participant. The study was conducted according to the guidelines of the Declaration of Helsinki.

### Statistical analysis

Data Analysis was performed statistical package of social science (SPSS 17.0) on windows 8.1. Arithmetic means and standard deviation were used. Test techniques used include, Independent student t-test and One-way ANOVA test one for comparing 2-independent groups and the other for more than 2-independent groups, Kruskalwallis and Mann–whitney test were used to compare more than 2-independent groups and to test the significance between groups. Sensitivity and specificity test were generated for testing a new test with ROC Curve (Receiver Operating Character). *p*-value < 0.05 was considered as a cutoff value for significance^44^. Deviations from Hardy–Weinberg disequilibrium (HWD) were calculated by chisquare(_2) goodness-of-fit test.

## Data Availability

All data generated or analyzed during this study are included in this article. Any further data required will be provided by Dr. Dalia A. Gaber.
